# Effects of Forest Age on Soil Autotrophic and Heterotrophic Respiration Differ between Evergreen and Deciduous Forests

**DOI:** 10.1371/journal.pone.0080937

**Published:** 2013-11-25

**Authors:** Wei Wang, Wenjing Zeng, Weile Chen, Yuanhe Yang, Hui Zeng

**Affiliations:** 1 Department of Ecology, College of Urban and Environmental Sciences, and Key Laboratory for Earth Surface Processes of the Ministry of Education, Peking University, Beijing, China; 2 Shenzhen Graduate School, Key Laboratory for Urban Habitat Environmental Science and Technology, Peking University, Shenzhen, China; 3 Institute of Botany, The Chinese Academy of Sciences, Beijing, China; Tennessee State University, United States of America

## Abstract

We examined the effects of forest stand age on soil respiration (SR) including the heterotrophic respiration (HR) and autotrophic respiration (AR) of two forest types. We measured soil respiration and partitioned the HR and AR components across three age classes ∼15, ∼25, and ∼35-year-old *Pinus sylvestris* var. *mongolica* (Mongolia pine) and *Larix principis-rupprechtii* (larch) in a forest-steppe ecotone, northern China (June 2006 to October 2009). We analyzed the relationship between seasonal dynamics of SR, HR, AR and soil temperature (ST), soil water content (SWC) and normalized difference vegetation index (NDVI, a plant greenness and net primary productivity indicator). Our results showed that ST and SWC were driving factors for the seasonal dynamics of SR rather than plant greenness, irrespective of stand age and forest type. For ∼15-year-old stands, the seasonal dynamics of both AR and HR were dependent on ST. Higher Q_10_ of HR compared with AR occurred in larch. However, in Mongolia pine a similar Q_10_ occurred between HR and AR. With stand age, Q_10_ of both HR and AR increased in larch. For Mongolia pine, Q_10_ of HR increased with stand age, but AR showed no significant relationship with ST. As stand age increased, HR was correlated with SWC in Mongolia pine, but for larch AR correlated with SWC. The dependence of AR on NDVI occurred in ∼35-year-old Mongolia pine. Our study demonstrated the importance of separating autotrophic and heterotrophic respiration components of SR when stimulating the response of soil carbon efflux to environmental changes. When estimating the response of autotrophic and heterotrophic respiration to environmental changes, the effect of forest type on age-related trends is required.

## Introduction

Forest soil respiration (SR) is the primary pathway where plant-fixed CO_2_ is released into the atmosphere [1]–[3]. This occurs from the root activity and their associated mycorrhizal fungi (belowground autotrophic respiration, AR) and from heterotrophic respiration (HR) [4], [5]. Quantifying forest SR, AR and HR components and their environmental controls requires an accurate evaluation of the response of the terrestrial carbon balance to future climate changes [8]–[10], based on the large annual exchange of carbon between forest ecosystems and the atmosphere [6], [7].

Forest age was reported to play an important role in determining the distribution of ecosystem carbon pools, fluxes and carbon sequestration [11]–[13]. Forest stands of various ages evolve with different abiotic and biotic environments that can differentially respond to environmental changes [14]. Older stands accumulate aboveground litter and root inputs, possibly decreasing HR and increasing AR [15]. Soil carbon dynamics may become more complex with increasing stand age [16]. For instance, the Great Lakes forest chronosequence showed that changes in soil carbon stocks across different aged stands had different patterns, with old-growth stands accumulating carbon in the deep soil layers, but not surface soils [16]. Our limited knowledge in forest succession and carbon cycles has resulted in few large-scale ecosystem carbon models accounting for the change of forest metabolic rates with age [17]. To model the long-term forest carbon dynamics and its coupling with the climate system, we need to understand the response of forest ecosystems to the changing climate, including the role of stand age and the successional status on carbon dynamics [16].

Previous studies on the effect of stand age on forest carbon efflux focused on the measurements of total SR in a single forest type. Researchers have observed that total SR increased [18], [19], decreased [15], [20], was similar [21], [22] or responded non-linearly [23], [24] with forest age. However, the effect of forest age on SR may depend on the forest type [25]. For instance, deciduous forests are time-limited each year to photosynthesize and allocate carbon to storage and reproduction compared with evergreen forests [26]. These physiological and phenological differences between deciduous and evergreen forests [27] may modulate the SR response across different aged stands to environmental changes. Additionally, AR and HR may respond differently to environmental changes [28]. AR is strongly influenced by photosynthetic activity compared with HR [28], [29], [30], [31]. Therefore, the ratio of AR and HR (of total SR) will modulate the response of SR across different aged stands to environmental changes [32], [33]. For instance, Jassal *et al.* (2012) observed that SR in younger stands (∼21 years) was more responsive to soil temperature (ST) and soil water content (SWC) compared with older stands (∼60 years) [34]. We measured SR and partitioned the HR and AR components across three age classes (∼15, ∼25, and ∼35 years) for the evergreen species, *Pinus sylvestris* var. *mongolica* (Mongolia pine) and the deciduous species, *Larix principis-rupprechtii* (larch) from June 2006 to October 2009 in a forest-steppe ecotone, northern China. We analyzed the relationships between SR, HR, AR and ST, SWC and NDVI (normalized difference vegetation index, indicative of plant greenness and net primary productivity). Our objective was to determine if forest type modulated the seasonal dynamics of SR and the AR and HR components and their responses to ST, SWC and NDVI across different aged stands. We tested the following hypotheses: (1) ST, SWC and NDVI will have significant effects on the seasonal dynamics of SR across varied aged stands, irrespective of forest type. This was based on the findings that SR depended on ST, SWC [35], [36] and primary productivity [29], [37]; (2) HR is more sensitive to ST and SWC than AR, irrespective of forest type. AR is controlled by NDVI, depending on stand age and forest type. The dependence of AR on NDVI was expected to decrease with stand age, because of the perennial life cycle, long carbon transport pathways and high storage capacity in older trees. However, AR dependence on NDVI may increase from deciduous to evergreen forest because of the greater dependence on seasonal accumulation and consumption of stored carbon in deciduous rather than evergreen forest [38].

## Materials and Methods

### Ethics Statement

The administration of the Saihanba Forestry Center gave permission for this research at each study site. We confirm that the field studies did not involve endangered or protected species.

### Site description

The study was conducted at Saihanba Forestry Center, Hebei Province, northern China (117°12′–117°30′ E, 42°10′–42°50′ N, 1400 m a.s.l.) adjacent to the Beijing-Tianjin region. Our study site lay within a typical forest-steppe ecotone in a temperate region. The climate is semi-arid, semi-humid, with long cold winters (November to March) and a short spring and summer. The annual mean air temperature and precipitation from 1964 to 2004 were −1.4°C and 450.1 mm, respectively. The soils are predominantly sandy, accompanied by meadow and marsh-type. The soil has low nutrient content with the organic carbon levels at 0.71–1.88% and total nitrogen at 0.08–0.19%. The bulk density ranged from 0.74 to 1.06 g cm^−3^ with litter and fine root biomass increasing with stand age (Table 1). Primary forests were harvested using large-scale industrial logging techniques in the late 1900s. This area has been threatened from sandstorm since the 1950s. Consequently, the China Forestry Administration proposed the establishment of a large plantation in Saihanba to ensure the environmental safety of the Beijing-Tianjin region. Saihanba forestry was established in 1962 with the plantation now 94,700 hm^2^, covering 76.8% of the area. This project has been the largest one in China with *P. sylvestris* var. *mongolica* (Mongolia pine) and *L. principis-rupprechtii* (larch) the most dominant species.

**Table 1:** Site characteristics of the six forest stands in this study.

Forest stands	Stand age (years)	Tree height (m)	DBH (cm)	Slope/aspect	Elevation (a.s.l.)	Soil texture	Tree density (stem ha^−1^)	SOC (%)	STN (%)	SBD (g cm^−3^)	LFB (g m^−2^)	FRB (g m^−2^)
*Larix principis-rupprechtii*	∼15	3.34	3.1	0°/-	1582	Sandy soil	1900	0.94	0.09	1.06	1360	191
	∼25	9.47	11.1	0°/-	1560	Sandy soil	2133	0.95	0.10	0.88	2207	198
	∼35	15.67	21.4	0°/-	1500	Sandy soil	1825	1.88	0.18	0.74	3412	220
*Pinus sylvestris* var. *mongolica*	∼15	2.70	4.3	1°/S	1509	Sandy soil	1800	0.71	0.08	0.98	1335	344
	∼25	8.02	12.0	2°/S	1519	Sandy soil	2010	1.27	0.13	0.83	2798	483
	∼35	14.70	20.7	1°/S	1502	Sandy soil	1760	1.60	0.19	0.85	2899	496

DBH =  Diameter at Breast Height (1.3 m); NDVI =  Normalized Difference Vegetation Index; SOC =  Soil Organic Carbon; STN =  Soil Total Nitrogen; SBD =  Soil Bulk Density; LFB =  Litter Floor Biomass; FRB =  Fine Root Biomass.

### Experimental design

**Figure 1:**
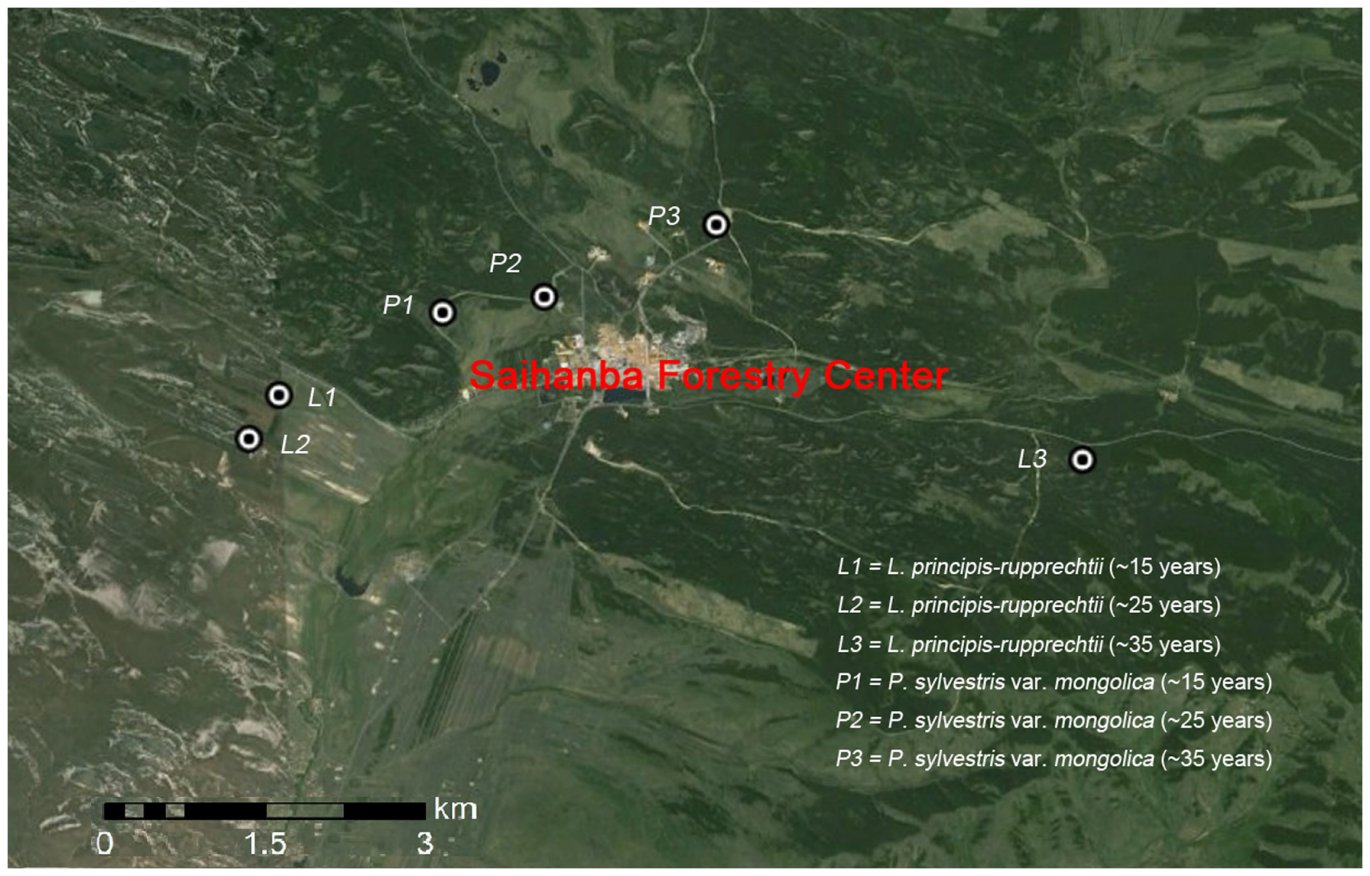
Location of sites showing different aged stands.

To assess the effects of forest age on soil respiration (SR), autotrophic respiration (AR) and heterotrophic respiration (HR) across different forest types, we selected six stands with the same planting density (4995 stem/ha), including three age classes of Mongolia pine and larch. These were pure plantations established on former meadow grassland dominated by *Leymus chinensis*. In combination, these stands made up a chronosequence from 15 to 35 years old, with the oldest stand ready for intermediate cutting. Neither fertilization nor drainage works had been carried out since tree establishment in any of the stands. Moreover, the topography at all six stands is predominantly flat with a similar slope, position and elevation (Table 1). The selected Mongolia pine plantation has a stand area of 1.25 ha (15 years old), 5 ha (25 years old) and 10.14 ha (35 years old). The selected larch plantation had a stand area of 1.05 ha (15 years old), 3.67 ha (25 years old) and 5.11 ha (35 years old). The locations of the stands with different stand ages are shown in Figure 1. Detailed stand information is shown in Table 1. For each stand, three replicate plots were arranged in an area of 20 m×20 m. Five subsamples (i.e. SR collars) were randomly arranged in each plot. The distance between any two stands was ≤10 km, avoiding differences in climate and soil type. Stand age was obtained from forest management records and from core samples using an increment borer. Similar climate and soil properties among these stands create an ideal chronosequence to study age effects on soil carbon efflux.

### Soil respiration (SR), soil temperature (ST) and water content (SWC)

SR was measured from June 2006 to October 2009 using a Li-8100 soil CO_2_ flux system (LI-COR Inc., Lincoln, NE, USA). During the growing season, five polyvinyl chloride (PVC) collars (10 cm inside diameter, 6 cm height) were inserted 3 cm into the soil in each plot and left *in situ* throughout the study. The five PVC collars were placed in each plot, one in each of the four corners and the fifth in the middle. Live plants inside the collars were clipped at the soil surface 1 day before each measurement. SR was measured every 15–20 days. To minimize the daily variation in SR and represent the daily mean, measurements were made between 0800 and 1100 h [39], [40]. For each measurement, the respiration rate was calculated as the mean of three plots per stand. During winter, longer soil collars (determined by snow depth, commonly >30 cm) were inserted into the soil surface and stabilized for 24 h before the SR measurement [41], [42]. The duration of winter at our site was 5 months from November to late March with near consistent, continuous mean daily soil temperature <0.5°C at 5 cm [43]. The Li-8100 soil CO_2_ flux system was kept in an isolated/heated container to maintain the temperature above freezing. Winter SR was measured monthly except in February.

During respiration measurements, ST was recorded in each collar at 5 cm soil depth with the Li-8100 temperature probe. Continuous measurements of ST were recorded at 30-min intervals with StowAway loggers (Onset Comp. Corp., Bourne, MA, USA) inserted in the soil at each site. SWC at 0–10 cm was measured inside the collars using time domain reflectometry (Soil moisture Equipment Corp., Santa Barbara, CA, USA). No data for SWC were obtained during the winter because the probe could not be fully inserted into the frozen soil.

### Harmonic analysis of time-series AVHRR normalized difference vegetation index (NDVI) dataset

NDVI is derived from the red: near-infrared reflectance ratio:
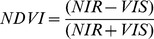
(1)where NIR and VIS represent the spectral reflectance measurements acquired in the near-infrared and visible (red) regions, respectively [44]. Due to their large areas, uniform distribution and sparse understory of both Mongolia pine and larch plantations, the NDVI of 16-Day L3Global 250 m product (MOD13 Q1) could well represent our plot measurements. We acquired the data from June 2006 to October 2009 using the website https://wist.echo.nasa.gov/api.

Harmonic (Fourier) analysis was used to remove the excess noise remaining in the NDVI time-series from satellite-borne data sources and to obtain reasonably smooth continuous daily data [45]. The Fourier series analysis decomposes a signal into an infinite series of harmonic components. Each of these components is composed initially of a sine wave and a cosine wave of equal integer frequency. These two waves are then combined into a single cosine wave with a characteristic amplitude (size of the wave) and phase angle (offset of the wave) [46], [47].

### Heterotrophic respiration (HR) and autotrophic respiration (AR)

To detect the response of HR and AR to environmental changes, five additional soil collars per plot were placed with deep PVC collars (80 cm^2^ and 70 cm deep) in October 2006. The 70-cm-long PVC collars isolated old plant roots, preventing new roots from growing inside the collars. This method has been successfully applied in various ecosystems to separate HR from the total SR [48]–[50].

To examine the transient response of dead root decomposition, we commenced the CO_2_ efflux measurements above the PVC tubes immediately after installation. The interference of installation was eliminated after 1.5 years (from 2006) with the soil CO_2_ efflux measured at the stands believed to represent HR. AR was calculated as the difference between SR and HR.

### SR, its HR and AR components and their relationships with ST, SWC and NDVI

We examined the relationships between SR, HR, AR and ST by fitting exponential functions to the data from each stand using the following equation:
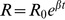
(2)where *R* is observed SR (plot-wide averages measured periodically throughout the year), *t* is the concurrent ST (5 cm depth), with *R_0_* being the basal respiration at temperature of 0°C and β the fitted parameters obtained using least squares nonlinear regression with SigmaPlot V. 8.02. The β values were used to calculate apparent Q_10_ values, which describes the change in respiration rate over a 10°C increase in *t* using:
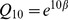
(3)


In addition, we calculated R_15_, the respiration values at a reference temperature of 15°C (without stimulation introduced by photosynthetic activity and without water limitations) [51] according to equation 4, using a Q_10_ coefficient calculated from equation (3). To remove the effect of ST, we examined the relationships between R_15_ and SWC and NDVI. 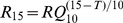
(4)where *R_15_* is the respiration flux at a constant temperature of 15°C, *R* the measured respiration rate, Q_10_ from equation (3) and *T* the ST at a 5 cm depth.

### Statistical analysis

To evaluate if SR, HR and AR significantly differed among different aged stands, forest type and measurement time, we used a three-way ANOVA. The relationships between SR, HR, AR and ST, SWC and NDVI were examined using regression analysis. All statistical analyses were performed with a significance level of 0.05 using SPSS (2009, ver. 18.0, SPSS Inc., Chicago, IL, USA).

## Results

### Seasonal dynamics of soil respiration (SR)

**Figure 2:**
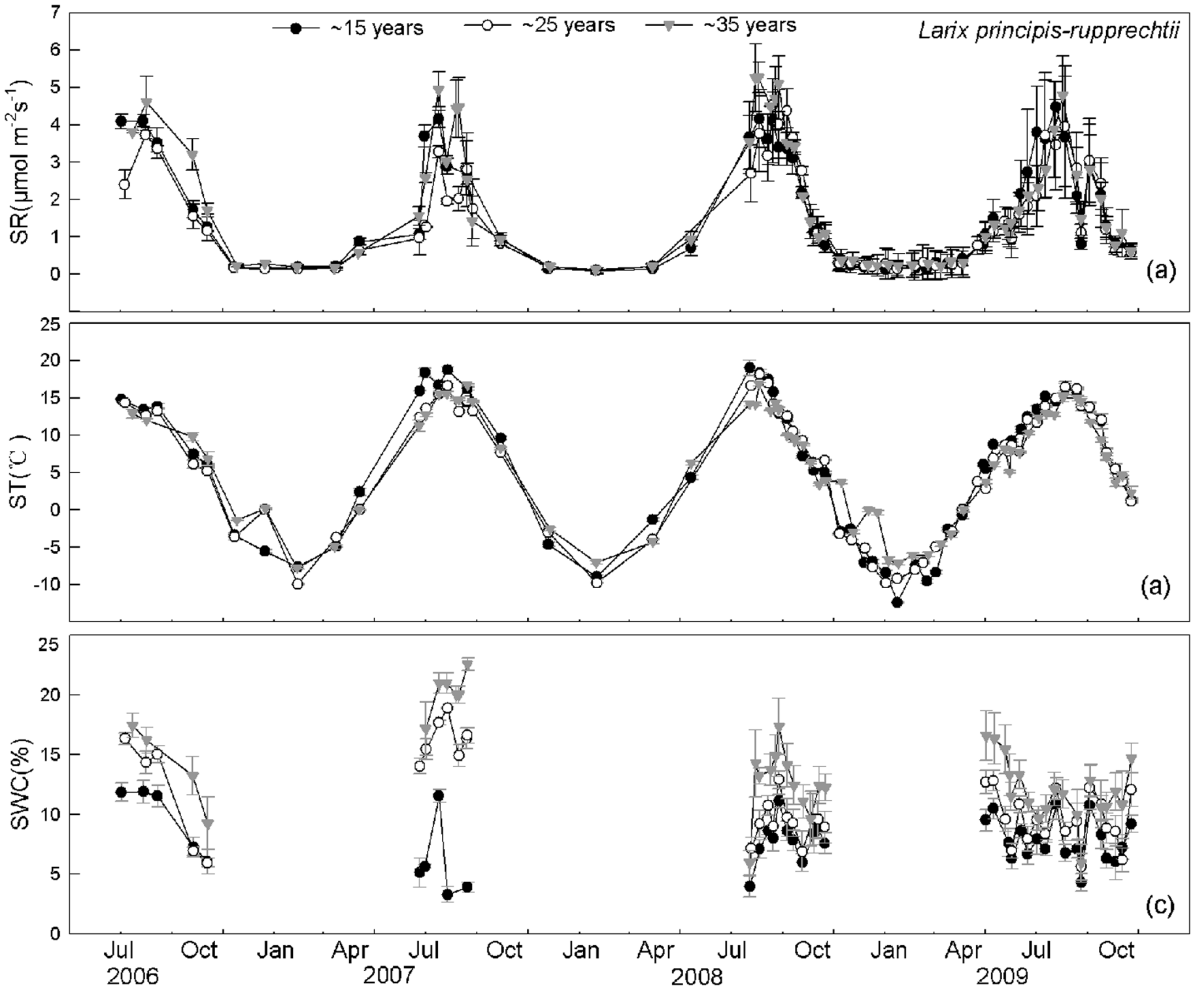
Seasonal changes of soil respiration (SR) and environmental factors across three stand ages of *Larix prinicipis*. SR (a), soil temperature at 5 cm depth (ST) (b) and soil water content at 10 cm depth (SWC) (c) are shown from June 2006 to October 2009.

**Figure 3:**
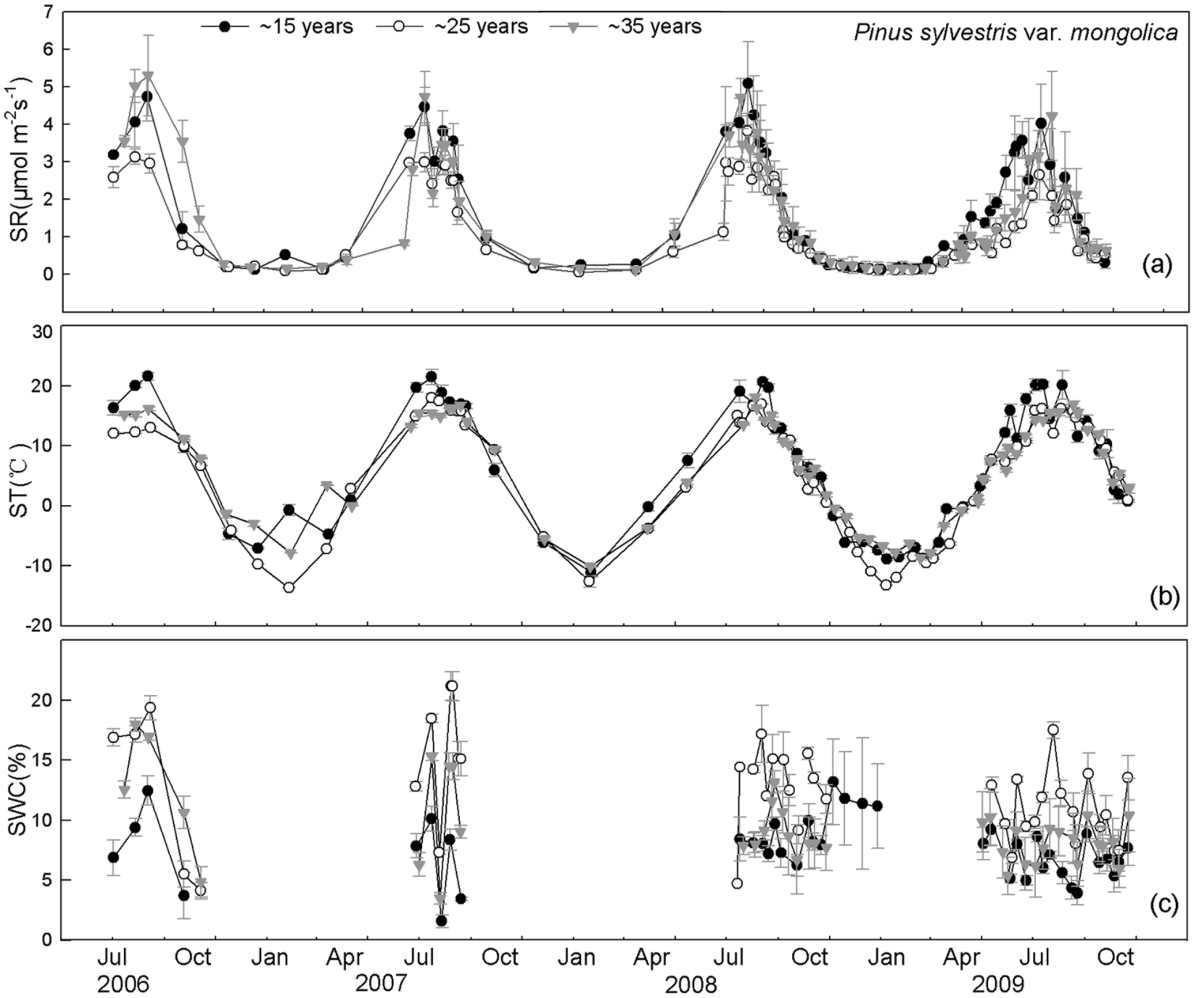
Seasonal changes of soil respiration (SR) and environmental factors across three stand ages of *Pinus sylvestris*. SR (a), soil temperature at 5 cm depth (ST) (b) and soil water content at 10 cm depth (SWC) (c) are shown from June 2006 to October 2009.

SR was significantly different among the different time measurements, irrespective of stand age and forest type (Table 2). SR was higher in summer and lower in winter, following the seasonal dynamics of ST, irrespective of stand age and forest type (Figs. 2, 3). Across different aged stands, the seasonal dynamics of SR were exponentially related to ST, possibly explaining the 88%–89% and 89%–95% variation in SR for larch and Mongolia pine, respectively (means for 4 years' measurements, Table 3). Apparent Q_10_ of SR across three stand ages ranged from 3.49–4.31 and 3.10–3.67 for larch and Mongolia pine, respectively (means of 4 years' measurements, Table 3). Apparent Q_10_ of ∼35-year-old stand was significantly higher compared with ∼15 and ∼25-year-old stands, irrespective of forest type (Table 3). Soil basal respiration (R_0_) was similar across three stand ages of both forest types (Table 3).

**Table 2:** Results of the three-way ANOVA with soil respiration (SR), heterotrophic respiration (HR) and autotrophic respiration (AR) as the response variables respectively, and stand age (∼15 years, ∼25 years and ∼35 years for SR, ∼15 years and ∼35 years for HR and AR), forest type (*Larix prinicipis vs. Pinus sylvestris*) and measurement time (from June 2006 to October 2009 for SR, from August 2008 to August 2009 for HR and AR) as factors.

Factor			SR					HR					AR		
	d.f.	Type III SS	Mean square	*F*	*P*	d.f.	Type III SS	Mean square	*F*	*P*	d.f.	Type III SS	Mean square	*F*	*P*
stand age	2	11.872	5.936	2.975	0.052	1	0.351	0.351	0.426	0.516	1	0.418	0.418	1.258	0.266
forest type	1	0.423	0.423	0.212	0.646	1	0.774	0.774	0.94	0.336	1	5.047	5.047	15.192	<0.01
time	3	34.367	11.456	5.741	<0.01	1	3.469	3.469	4.211	<0.05	1	1.289	1.289	3.879	0.053
stand age × forest type	2	2.071	1.035	0.519	0.596	1	0.012	0.012	0.015	0.904	1	2.344	2.344	7.056	<0.01
stand age × time	6	4.808	0.801	0.402	0.878	1	1.202	1.202	1.459	0.231	1	0.338	0.338	1.017	0.317
forest type × time	3	3.716	1.239	0.621	0.602	1	0.064	0.064	0.078	0.781	1	1.336	1.336	4.021	<0.05
stand age × forest type × time	6	2.39	0.398	0.2	0.977	1	0.771	0.771	0.935	0.337	1	0.11	0.11	0.332	0.567

The data in ∼25-year-old stands were absent because of the artificially damaged collars for separating AR and HR during the experiments.

**Table 3:** Q_10_ of soil respiration (SR), basal respiration rate (R_0_) and determination coefficient of exponential relationships between SR and soil temperature (ST), using *SR* = *R_0_*×*e^βT^*. L1, L2, L3 are ∼15 years, ∼25 years and ∼35 years *Larix prinicipis* stand, respectively.

	2006				2007				2008				2009				Mean values for 4 years			
	R_0_	β	Q_10_	R^2^	R_0_	β	Q_10_	R^2^	R_0_	β	Q_10_	R^2^	R_0_	β	Q_10_	R^2^	R_0_	β	Q_10_	R^2^
L1	0.40	0.17	5.37	0.99	0.39	0.11	3.06	0.90	0.43	0.14	4.06	0.91	0.48	0.12	3.25	0.90	0.45	0.13	3.53	0.89
L2	0.29	0.18	6.30	0.87	0.37	0.12	3.22	0.91	0.49	0.14	3.97	0.94	0.50	0.11	3.10	0.90	0.45	0.13	3.49	0.88
L3	0.31	0.22	8.94	0.98	0.40	0.14	3.86	0.91	0.41	0.18	5.87	0.92	0.52	0.13	3.56	0.93	0.46	0.15	4.31	0.89
P1	0.35	0.13	3.49	1.00	0.38	0.12	3.35	0.97	0.50	0.12	3.25	0.94	0.42	0.11	3.10	0.95	0.43	0.12	3.19	0.95
P2	0.45	0.12	3.39	0.85	0.37	0.12	3.29	0.97	0.46	0.12	3.46	0.91	0.35	0.10	2.72	0.91	0.40	0.11	3.10	0.89
P3	0.36	0.17	5.53	0.98	0.38	0.12	3.39	0.85	0.50	0.14	3.94	0.94	0.39	0.12	3.35	0.94	0.42	0.13	3.67	0.90

P1, P2, P3 are ∼15 years, ∼25 years and ∼35 years *Pinus sylvestris* stand, respectively.

SR_15_ showed no significant relationship with NDVI, irrespective of forest age and type (*P*>0.05 for all stands, Fig. S1). Conversely, a significant correlation was found between SR_15_ and SWC, which could explain the variation of 15%–26% and 10%–33% variation for larch and Mongolia pine, respectively (Fig. S2).

### Seasonal dynamics of heterotrophic respiration (HR) and autotrophic respiration (AR)

**Figure 4:**
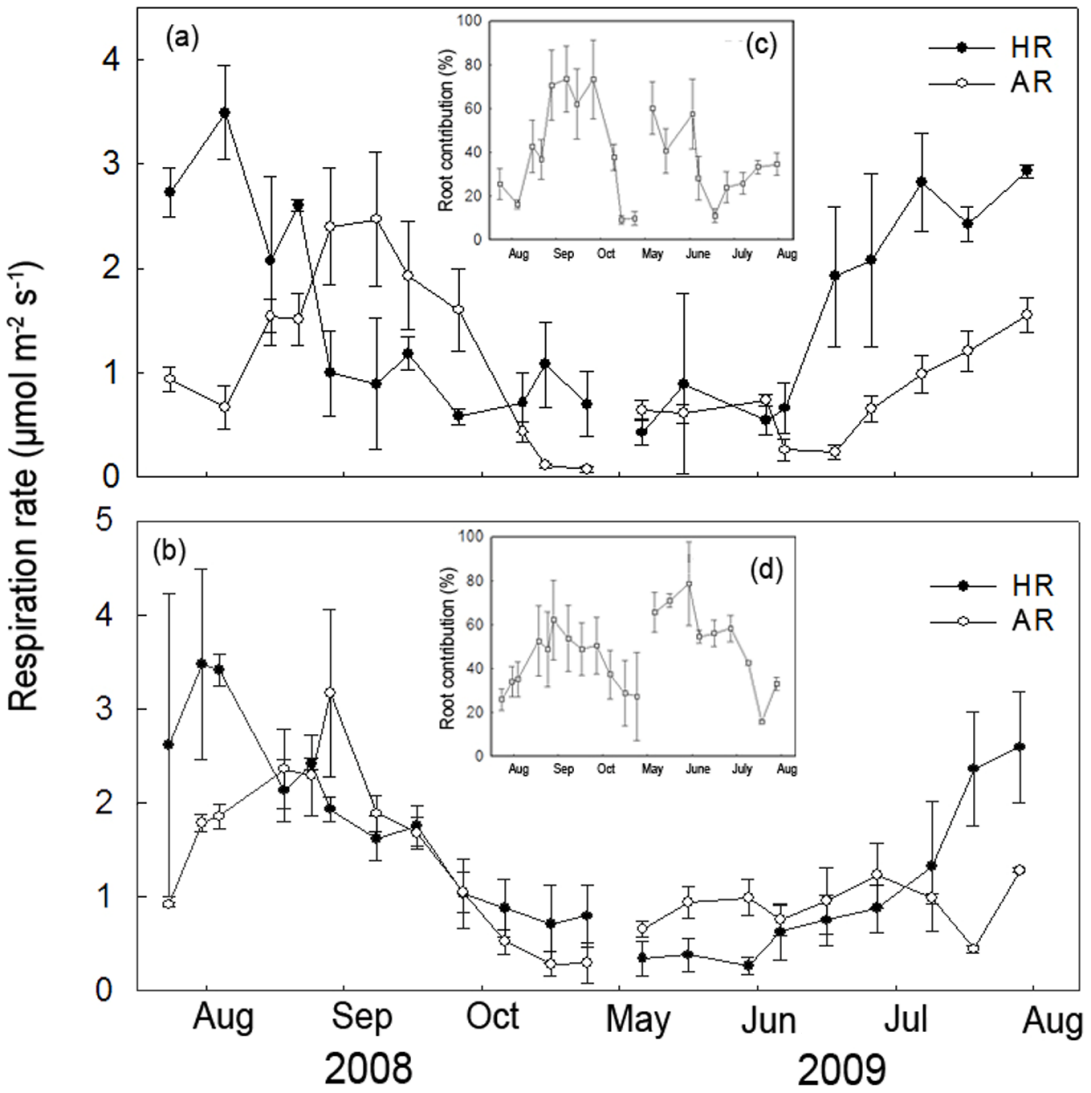
Seasonal changes in autotrophic and heterotrophic components of soil respiration (SR) for different aged stands of *Larix prinicipis*. Autotrophic respiration (AR) and heterotrophic respiration (HR) in ∼15-year-old (a) and ∼35-year-old stands (b) are shown from August 2008 to August 2009. Seasonal changes in the contribution of AR to SR are shown in inserted figures for ∼15-year-old (c) and ∼35-year-old (d) stands. The data in the ∼25-year-old stand were absent because of the artificially damaged collars for separating AR and HR during the experiments.

**Figure 5:**
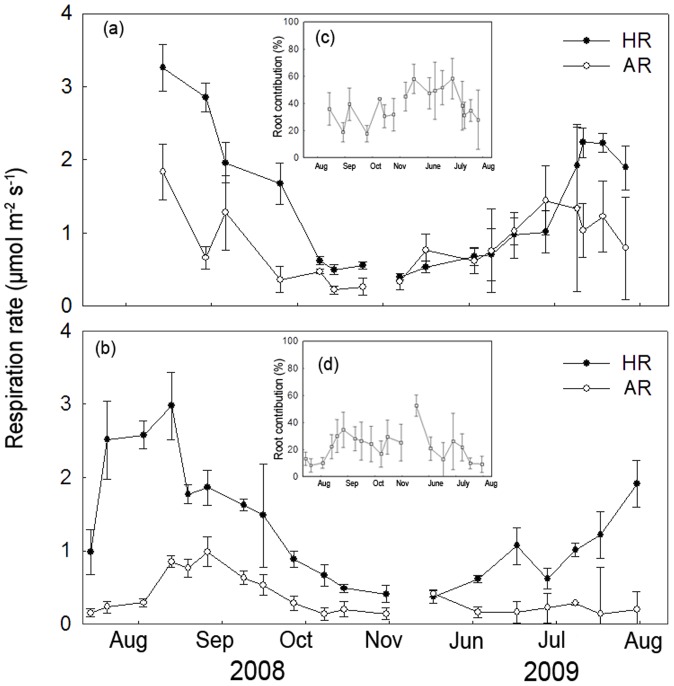
Seasonal changes in autotrophic and heterotrophic components of soil respiration (SR) for different aged stands of *Pinus sylvestris*. Autotrophic respiration (AR) and heterotrophic respiration (HR) in ∼15-year-old (a) and ∼35-year-old stands (b) are shown from August 2008 to August 2009. Seasonal changes in the contribution of AR to SR are shown in inserted figures for ∼15-year-old (c) and ∼35-year-old (d) stands. The data in the ∼25-year-old stand were absent because of the artificially damaged collars for separating AR and HR during the experiments.

HR was significantly different among different time measurements, irrespective of stand age and forest type (Table 2). The seasonal dynamics of HR were similar across different aged stands, showing higher values in summer and lower in spring and autumn (Figs. 4, 5). However, AR showed a more variable pattern. For larch, maximal AR occurred in early September with minimal values in late October, irrespective of stand age (Fig. 4a, 4b). The contribution of AR to total SR ranged from 9%–74% and 16%–79% (∼15 and ∼35-year-old-stands, respectively; Fig. 4c and d). HR, AR and the contribution of AR to total SR showed no significant differences between ∼15 and ∼35-year-old larch (*P*>0.05 for all the comparisons, Table 2). For Mongolia pine, AR peaked in early August and late August (∼15 and ∼35-year-old-stands, respectively), with minimum AR in late October for both stands (Fig. 5a, 5b). The contribution of AR to total SR ranged from 16%–63% (Fig. 5c) for ∼15 and 8%–79% for ∼35-year-old stands (Fig. 5d). AR and the contribution of AR to total SR was significantly lower in the ∼35 compared with ∼15-year-old stand (39% *vs.* 22%; *P*<0.05). HR showed no significant differences between the two stands (*P*>0.05, Table 2). AR was significantly different between the two forest types, with a significant interaction effect among forest type, stand age and measurement time (Table 2).

**Figure 6:**
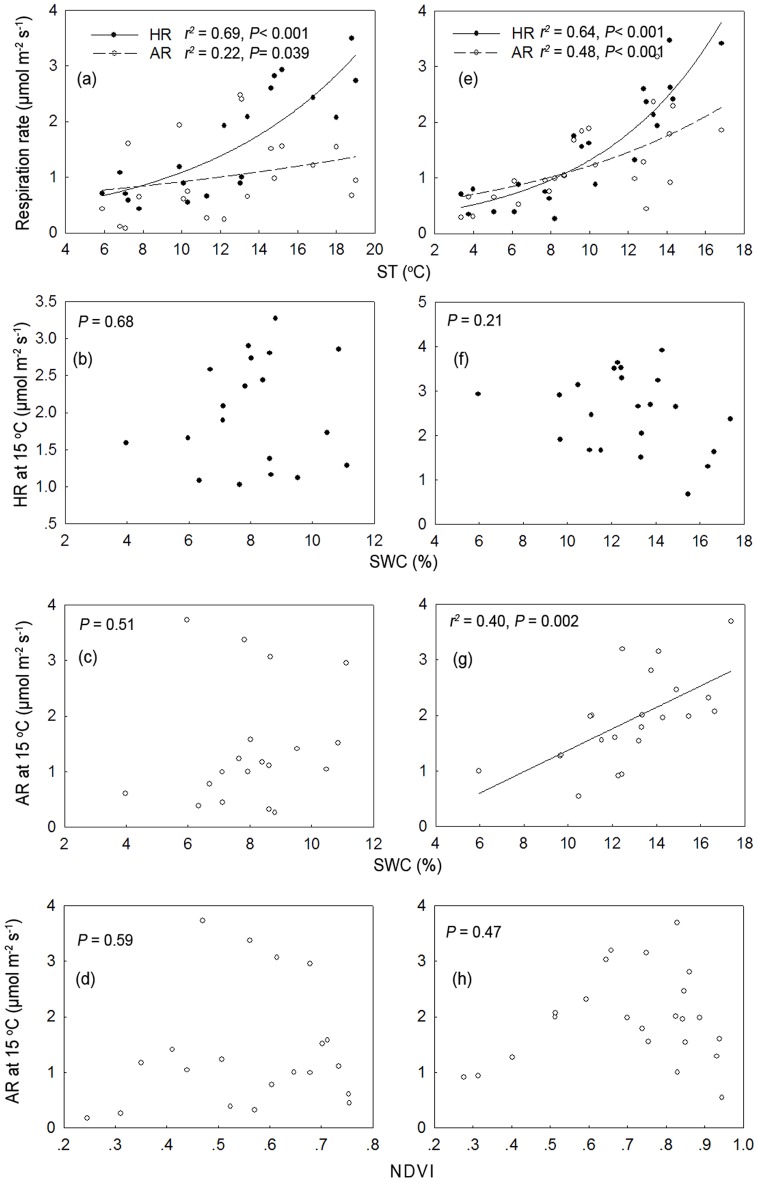
Relationships between autotrophic respiration (AR) and heterotrophic respiration (HR) and environmental factors across different aged stands of *Larix prinicipis*. The relationships are shown between autotrophic respiration (AR) and heterotrophic respiration (HR) and soil temperature (ST) for ∼15-year-old (a), ∼35-year-old stands (e), between soil water content (SWC) and HR_15_ for ∼15-year-old (b), ∼35-year-old stands (f), between SWC and AR_15_ for ∼15-year-old (c) and ∼35-year-old stands (g), and between NDVI and AR_15_ for ∼15-year-old (d), and ∼35-year-old (h) stands of *Larix prinicipis*. The data in the ∼25-year-old stand were absent because of the artificially damaged collars for separating AR and HR during the experiments.

**Figure 7:**
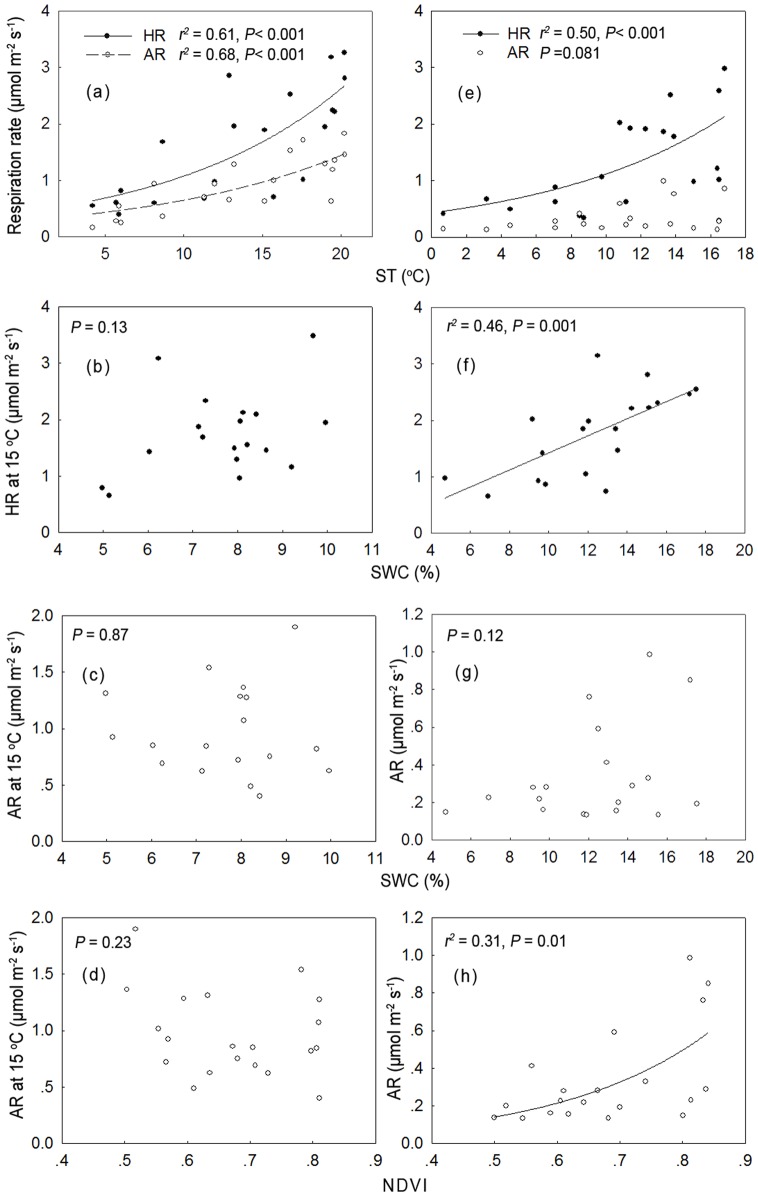
Relationships between autotrophic respiration (AR) and heterotrophic respiration (HR) and environmental factors across different aged stands of *Pinus sylvestris*. The relationships are shown between autotrophic respiration (AR) and heterotrophic respiration (HR) and soil temperature (ST) for ∼15-year-old (a), ∼35-year-old stands (e), between soil water content (SWC) and HR_15_ for ∼15 years (b) and ∼35 years (f); between SWC and AR_15_ for ∼15-year-old (c) and ∼35-year-old stands (g); between NDVI and AR_15_ for ∼15-year-old stand (d), and between NDVI and AR for ∼35-year-old stands (h) of *Pinus sylvestris*. The data in the ∼25-year-old stand were absent because of the artificially damaged collars for separating AR and HR during the experiments.

The seasonal dynamics of AR and HR were dependent on ST in the ∼15-year-old stand, which could explain the 22% and 69% variation in AR and HR, respectively for larch (Fig. 6a–6d). Additionally, this could explain the 68% and 61% variation in AR and HR (respectively) for Mongolia pine (Fig. 7a–7d). We found a higher Q_10_ for HR compared with AR in larch (3.85 *vs.* 2.98). In contrast, Mongolia pine showed a similar Q_10_ for HR compared with AR (2.61 *vs.* 2.76). As the stand age increased, the Q_10_ of both HR (3.85, ∼15-year-old *vs.* 5.07, ∼35-year-old) and AR (2.98, ∼15-year-old *vs.*3.23, ∼35 years) increased in larch (Fig. 5a, 5b). For Mongolia pine, the Q_10_ of HR increased (2.61, ∼15-year-old *vs*. 2.71, ∼35-year-old), but AR showed no significant relationship with ST in ∼35-year-old stand (Fig. 7e). In ∼15-year-old stand, neither HR nor AR was controlled by SWC, irrespective of forest type. As stand age increased, HR correlated with SWC in Mongolia pine (Fig. 7f). In contrast, larch AR was significantly related with SWC (*P*<0.01, Fig. 6g). The dependence of AR on NDVI only occurred in ∼35-year-old Mongolia pine (Fig. 6d, 6h, Fig. 7d, 7h).

## Discussion

### Drivers of seasonal dynamics for soil respiration (SR)

We hypothesized (1) that ST, SWC and NDVI will impose significant effects on the seasonal dynamics of SR across different aged stands, irrespective of forest type. However, we found that the seasonal dynamics of SR were principally driven by ST and SWC, not by NDVI, across different aged stands, irrespective of forest type. Forest SR is closely related to gross primary productivity (GPP) and leaf area index (LAI) [52], [53], suggesting a coupling between the CO_2_ assimilation in the forest canopy and CO_2_ released from the soil. NDVI has shown a close correlation with GPP [54], [55] and LAI [56]. As a convenient proxy for productivity, NDVI was expected to be positively associated with AR because of the dependence on photosynthesis [52]. We expected a positive relationship between SR and NDVI based on the higher contribution of AR to SR (Figs. 4, 5). We found strong positive relationships between SR and NDVI (Fig. S3); however, these relationships disappeared when we used R_15_ for both forest types across three different aged stands (Fig. S1). These results imply that the correlation between SR and NDVI may occur because of the significant relationship between ST and NDVI (Fig. S4). Our results emphasized the significance of ST and SWC on simulating total SR for both forest types across different aged stands. Our results were inconsistent with recent evidence showing that SR was closely related to canopy photosynthesis at various timescales [57], [58]. The discrepancies may occur because NDVI measures integrated past photosynthetic activity rather than present photosynthetic activity [59]. In addition, differences may have occurred because the seasonal change in foliar photosynthetic capacity and NDVI are not necessarily coincidence [53]. Moreover, the temporal dynamics of SR were reported to lag behind those of NDVI [33], [60], so our monthly SR measurements may not reflect the real relationship between these two variables. Therefore, high-frequency measurements should be conducted to explore the control of temporal dynamics in SR.

### Drivers of seasonal dynamics of heterotrophic respiration (HR) and autotrophic respiration (AR)

In our second hypothesis, we expected greater sensitivity in HR to variation in ST compared with AR. However, we observed a mixed result. In ∼15-year-old stand, we found a higher Q_10_ for HR compared with AR in larch and a similar Q_10_ for AR with HR in Mongolia pine (Fig. 6a, 7a). The lower Q_10_ of AR compared with HR in larch may be attributed to differing plant physiology and phenology in Mongolia pine. Deciduous trees are time-limited each year to photosynthesize, allocating more carbon for aboveground growth during summer when ST is highest [26]. Furthermore, peak root growth commonly occurs in spring and autumn for coniferous forests [61], [62]. We observed the contribution of AR to total SR was higher in spring and autumn for larch. Therefore, the seasonal dynamics of AR in larch may be connected with those of root growth, resulting in lower Q_10_ of AR compared with HR. In contrast, Mongolia pine can photosynthesize year-round with little investment required in highly efficient photosynthetic activity. Consequently, the dynamics of AR in Mongolia pine stands may be associated with higher metabolic demand during leaf production [63]. We also found maximal AR in early summer in Mongolia pine (Fig. 5a), which was consistent with Lee *et al.*(2010), who observed maximal contribution of AR to total SR in summer for evergreen forest and in autumn for deciduous forest [64].

According to our second hypothesis, we expected that the seasonal dynamics of AR were more dependent on NDVI rather than HR. However, we found that AR was significantly affected by NDVI only in ∼35-year-old Mongolia pine (Fig. 7h). We did not observe a significant relationship between AR and NDVI in larch, possibly because of greater carbon allocation in deciduous forest to storage and reproductive functions compared with evergreen forests [26]. The relationship between AR and NDVI in deciduous trees was more dependent on seasonal accumulation and consumption of stored C compared with evergreen trees [65]. The absence of a significant relationship in ∼15-year-old Mongolia pine stand may be attributed to the soil water deficit, inducing a selection pressure favoring trees presenting high storage capability for reserve compounds to combat this stress [66], [67]. Moreover, the carbon balance between growth and storage remained constant between age classes in evergreen forest [26]. Our results suggested that the correlation between AR and NDVI was dependent upon stand age, and this stand age-related effect was forest type-dependent.

We expected a higher dependence of HR on SWC compared with AR because AR was reported as less responsive to water variability compared with HR [68], [69]. However, we found in ∼15-year-old stand that neither HR nor AR was controlled by SWC, irrespective of forest type. This may occur because of the narrower variation in SWC in young forest characterized by high soil water evaporation [70]. With increased stand age, HR correlated with SWC in Mongolia pine (Fig. 7f). In contrast, larch AR was significantly related to SWC (Fig. 6g). Similar observations were reported by Lee *et al.* (2010), who found a significant correlation between AR and SWC in *Quercus*-dominated deciduous stand, not evergreen stands of *Abies holophylla*. One possible reason for this difference between Mongolia pine and larch in the dependence of AR and HR on SWC may be associated with reduced root longevity of deciduous tree species (<1 year) compared with evergreen species (<1–12 years) [71], [72]. Higher turnover rates in deciduous tree roots may induce a rapid change in response to environmental fluctuations compared with evergreen trees. Therefore, our results suggest the response of AR and HR to SWC depended on stand age, with the effects of stand age dependent on forest type.

## Conclusions

In summary, we tested the effects of forest type on SR, AR and HR across different aged stands in the same location to prevent confounding from climatic and edaphic conditions. We observed that ST and SWC were significant factors controlling the seasonal dynamics of total SR, irrespective of forest age and type. However, the response of AR and HR to ST, SWC and NDVI differed across various aged stands, with the effects of stand age dependent on the forest type. These results suggest that in stimulating the response of forest carbon efflux to environmental changes, we should consider the effects of stand age and the influence of forest type on this age-related trend. Furthermore, our study emphasized considering the response of HR and AR to environmental changes separately when predicting the response of soil carbon efflux in different aged forests to global climate changes. Future research should attempt an in-depth understanding of the effects of more functional types on carbon efflux across different aged stands.

## Acknowledgments

We thank two anonymous reviewers for their constructive suggestions on an early version of this manuscript.

## Supporting Information

Figure S1 Relationships between soil respiration at 15°C (SR_15_) and NDVI across different aged stands. The relationships are shown for ∼15 (a), ∼25 (b), ∼35-year-old stands (c) of *Larix prinicipis*, ∼15 (d), ∼25 (e), and ∼35 year-old stands (f) of *Pinus sylvestris*.
(TIF)

Figure S2 Relationships between soil respiration at 15°C (SR_15_) and soil water content (SWC) across different aged stands. The relationships are shown for ∼15 (a), ∼25 (b) and ∼35-year-old stands (c) of *Larix prinicipis*, ∼15 (d), ∼25 (e) and ∼35- year-old stands (f) of *Pinus sylvestris*.
(TIF)

Figure S3 Relationships between soil respiration (SR) and NDVI across different aged stands. The relationships are shown for ∼15 (a), ∼25 (b) and ∼35- year-old stands (c) of *Larix prinicipis*, ∼15 (d), ∼25 (e) and ∼35-year-old stands (f) of *Pinus sylvestris*.
(TIF)

Figure S4 Relationships between ST (soil temperature at 5 cm depth) and NDVI for non-growing season (dotted line) and growing season (solid line). The relationships are shown for ∼15 (a) and ∼25-year-old stands (b) of *Larix prinicipis*, ∼15 (c), ∼25 (d), and ∼35-year-old stands (e) of *Pinus sylvestris*. The data of ∼35-year-old stands *L. prinicipis* were absent because of the damage of StowAway loggers inserted in the soil.
(TIF)
