# Supplementary figures and images for: Effects of Forest Age on Soil Autotrophic and Heterotrophic Respiration Differ between Evergreen and Deciduous Forests
Source: PLoS One. 2013 Nov 25;8(11):e80937. doi: 10.1371/journal.pone.0080937 (PMC3839927; doi:10.1371/journal.pone.0080937)

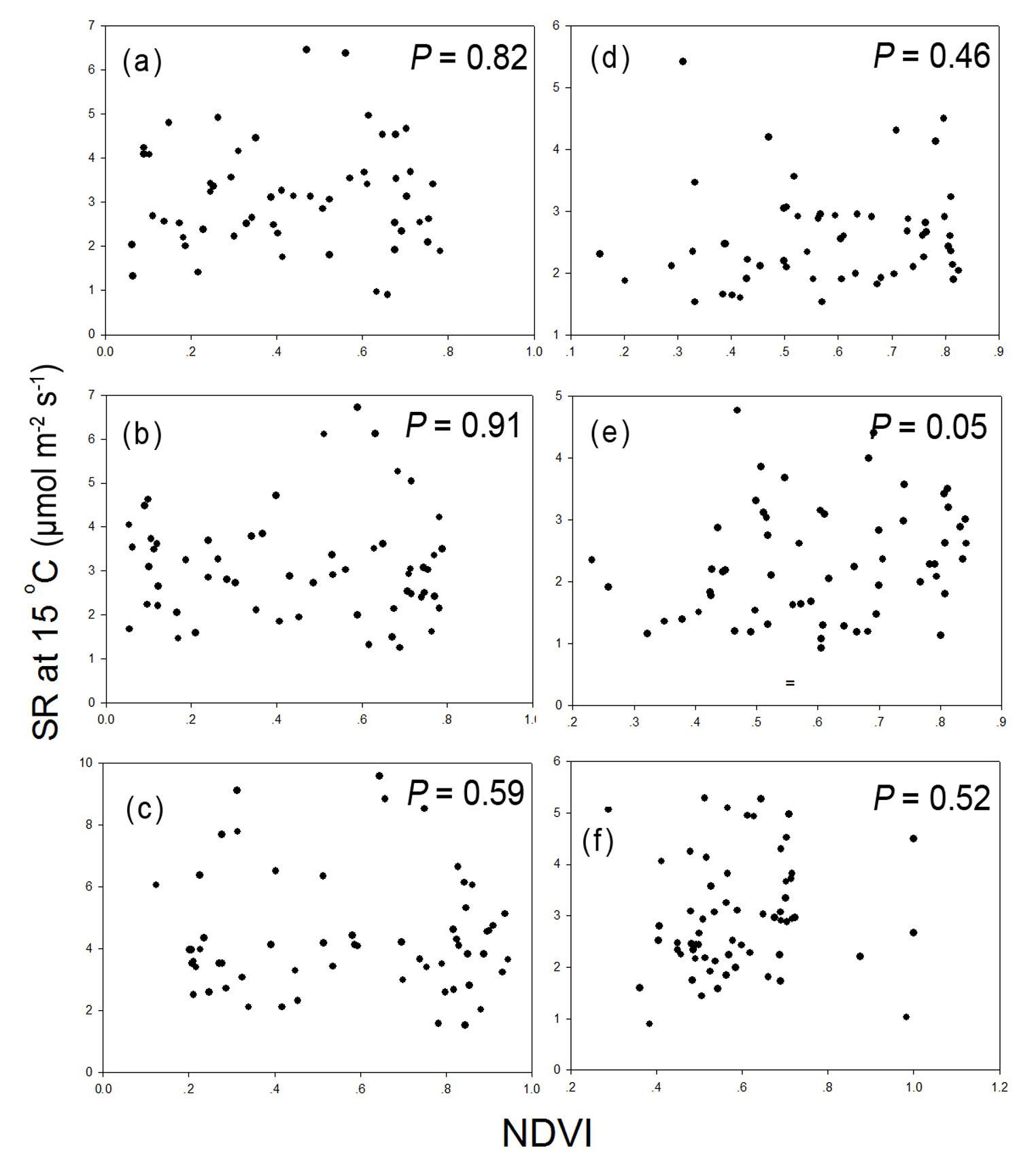

Supplement: Figure S1 — Relationships between soil respiration at 15°C (SR15) and NDVI across different aged stands. The relationships are shown for ∼15 (a), ∼25 (b), ∼35-year-old stands (c) of Larix prinicipis, ∼15 (d), ∼25 (e), and ∼35 year-old stands (f) of Pinus sylvestris. (TIF) [file pone.0080937.s001.tif]

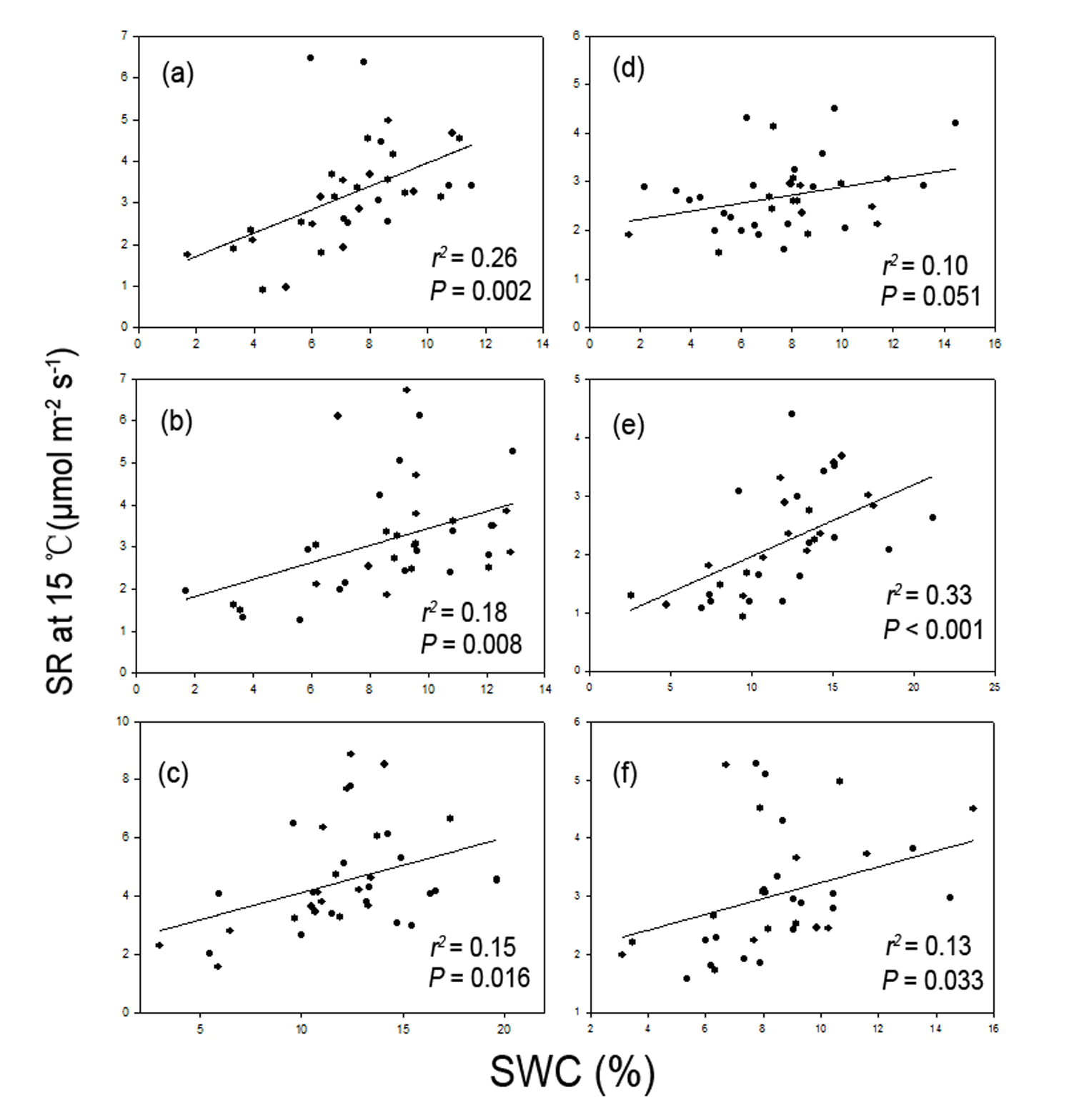

Supplement: Figure S2 — Relationships between soil respiration at 15°C (SR15) and soil water content (SWC) across different aged stands. The relationships are shown for ∼15 (a), ∼25 (b) and ∼35-year-old stands (c) of Larix prinicipis, ∼15 (d), ∼25 (e) and ∼35- year-old stands (f) of Pinus sylvestris. (TIF) [file pone.0080937.s002.tif]

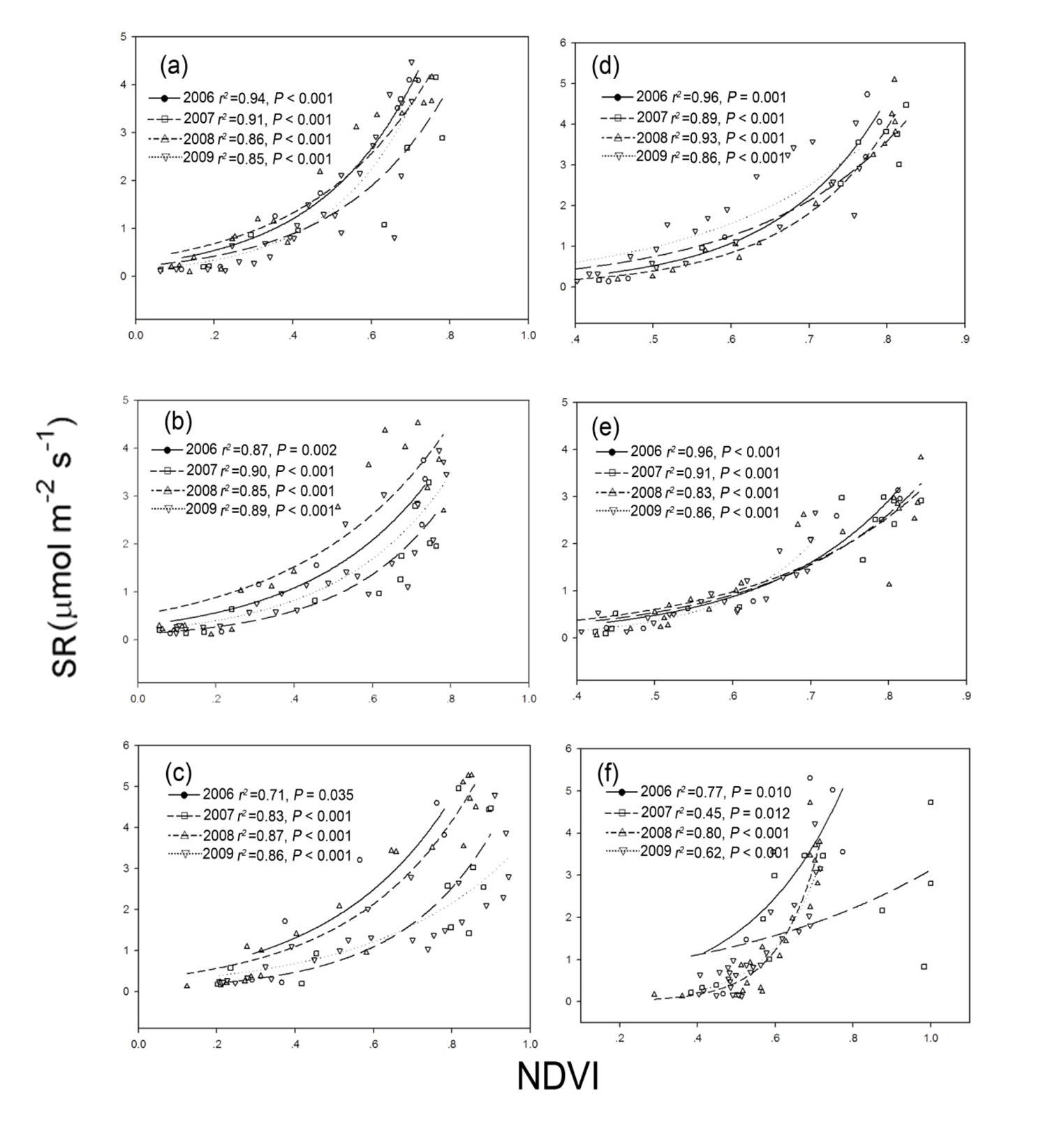

Supplement: Figure S3 — Relationships between soil respiration (SR) and NDVI across different aged stands. The relationships are shown for ∼15 (a), ∼25 (b) and ∼35- year-old stands (c) of Larix prinicipis, ∼15 (d), ∼25 (e) and ∼35-year-old stands (f) of Pinus sylvestris. (TIF) [file pone.0080937.s003.tif]

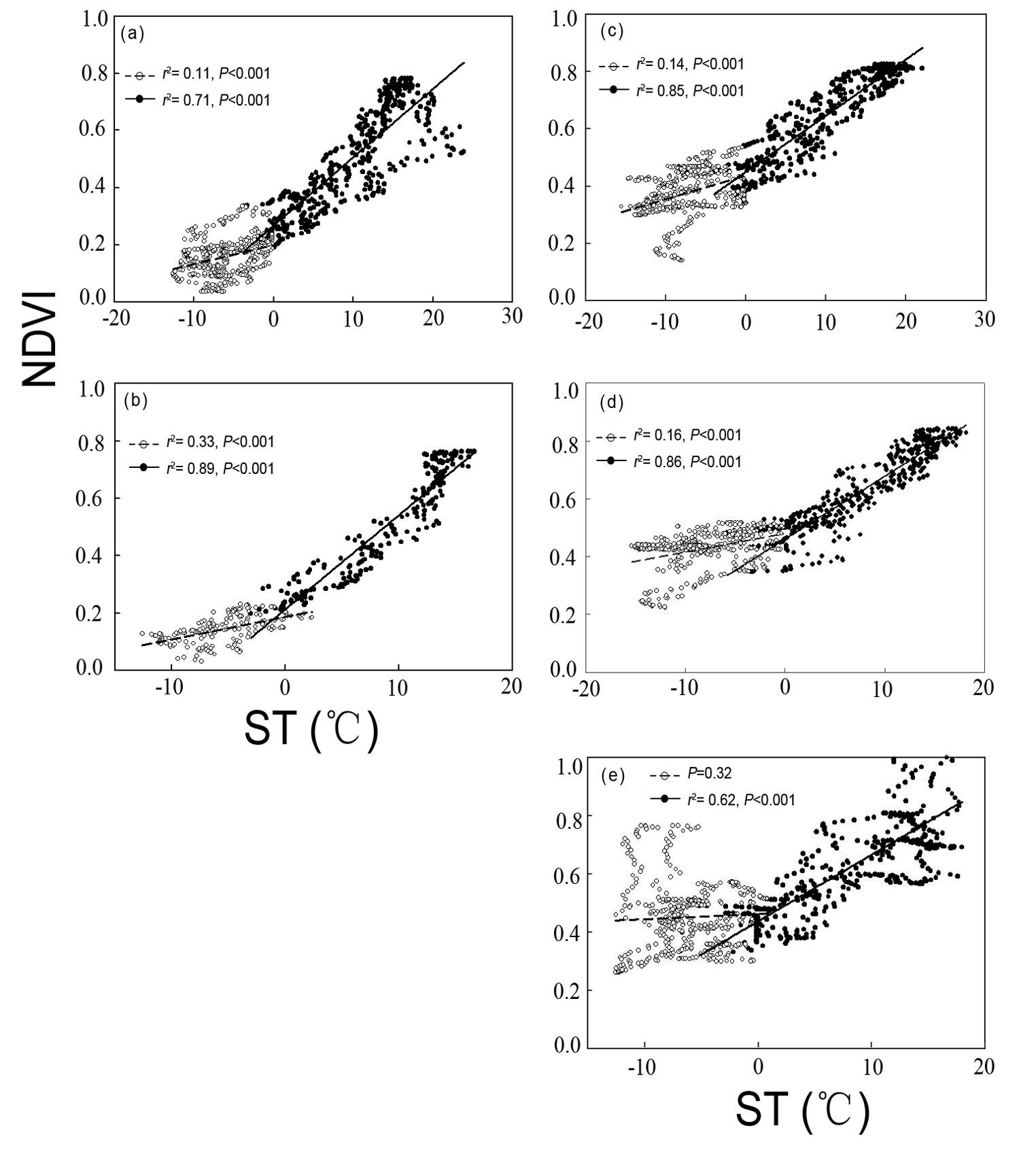

Supplement: Figure S4 — Relationships between ST (soil temperature at 5 cm depth) and NDVI for non-growing season (dotted line) and growing season (solid line). The relationships are shown for ∼15 (a) and ∼25-year-old stands (b) of Larix prinicipis, ∼15 (c), ∼25 (d), and ∼35-year-old stands (e) of Pinus sylvestris. The data of ∼35-year-old stands L. prinicipis were absent because of the damage of StowAway loggers inserted in the soil. (TIF) [file pone.0080937.s004.tif]
